# Chronic lung diseases and kidney disease: pathophysiology and management

**DOI:** 10.1093/ndt/gfaf077

**Published:** 2025-04-29

**Authors:** Silvia De Rosa, Sergio Lassola, Fabio Silvio Taccone, Denise Battaglini

**Affiliations:** Centre for Medical Sciences - CISMED - Interdepartmental Center for Medical Sciences, University of Trento, Trento, Italy; Department of Anesthesia and Intensive Care, Santa Chiara Hospital, APSS Trento, Trento, Italy; Department of Anesthesia and Intensive Care, Santa Chiara Hospital, APSS Trento, Trento, Italy; Department of Intensive Care, Hôpital Universitaire de Bruxelles (HUB), Université Libre de Bruxelles (ULB), Brussels, Belgium; Department of Anesthesia and Intensive Care, IRCCS Ospedale Policlinico San Martino, Genova, Italy

**Keywords:** acute kidney injury, chronic kidney disease, chronic lung disease, hypoxemia, pulmonary-renal cross-talk

## Abstract

Acute kidney injury (AKI) is a critical complication in patients with chronic lung diseases (CLD), particularly during acute exacerbations. This review focuses on the pathophysiological mechanisms linking CLD to AKI and highlights key clinical strategies to mitigate its impact. CLD patients with pre-existing kidney dysfunction face an increased risk of AKI due to impaired gas exchange, systemic inflammation, and neurohormonal activation. Hypoxemia and hypercapnia contribute to kidney hypoperfusion, endothelial dysfunction, and sodium–water imbalances, exacerbating renal injury. Current management strategies prioritize minimizing mechanical ventilation-related damage, optimizing hemodynamics, and preventing AKI progression. A multidisciplinary approach is essential to improving outcomes, emphasizing early identification and targeted interventions for CLD-associated AKI.

## INTRODUCTION

Patients with chronic lung diseases (CLD) are at a heightened risk of acute kidney injury (AKI), particularly during acute exacerbations. While the connection between CLD and chronic kidney disease (CKD) is well recognized, AKI represents a more immediate and severe consequence of lung dysfunction [1, 2]. In addition to this well-established connection, several risk factors such as CKD, tobacco smoking, and obesity play a pivotal role in predisposing patients with CLD to AKI [3]. CKD is a strong predictor of AKI risk due to pre-existing alterations in renal hemodynamics and reduced functional reserve [4]. Similarly, tobacco smoking and obesity are recognized contributors to both chronic lung and kidney diseases through mechanisms involving systemic inflammation, oxidative stress, and vascular dysfunction [5]. These shared risk factors highlight the need for integrated prevention strategies and underscore the importance of early identification of at-risk populations [2].

This review aims to clarify the key mechanisms by which hypoxemia, hypercapnia, and systemic inflammation in CLD contribute to AKI development [6–9]. Episodes of acute respiratory failure in CLD patients can precipitate AKI through a combination of hypoxemia, hypercapnia, systemic inflammation, and altered hemodynamics [10].

The pathophysiological link between CLD and AKI is driven by multiple mechanisms. While lung ventilation has traditionally been considered a risk factor for kidney injury, recent evidence suggests that when hemodynamics is well maintained, both acute and chronic lung ventilation may have a minimal injurious effect on kidney function [11]. Hypoxemia leads to reduced oxygen delivery to the kidney, exacerbating tubular ischemia and impairing glomerular filtration [12]. Hypercapnia, through its vasoconstrictive effects, increases renovascular resistance, reducing renal perfusion. Additionally, systemic inflammation and neurohormonal activation further compromise kidney function, contributing to sodium retention, endothelial dysfunction, and fluid overload [13]. These interactions create a vicious cycle where lung dysfunction exacerbates kidney injury, which in turn worsens respiratory outcomes [2].

Beyond common CLD presentations, certain patient populations exhibit an increased susceptibility to kidney dysfunction. However, a detailed discussion of these specific populations falls outside the scope of this review. For instance, individuals with cystic fibrosis frequently experience renal complications due to chronic infections, nephrotoxic antibiotic exposure, and metabolic imbalances [14]. Similarly, lung transplant recipients are prone to kidney disease because of long-term immunosuppressive therapy and perioperative complications. These unique cases further highlight the importance of kidney monitoring in patients with chronic or progressive lung diseases [15].

Given the significant morbidity and mortality associated with AKI in CLD patients, there is a critical need for focused efforts to diagnose and manage this condition effectively [16–18].

Such efforts are essential not only for immediate care but also for preventing the progression from kidney dysfunction to end-stage kidney disease [19]. Research into the pathophysiological mechanisms connecting CLD to kidney impairment has started to shed light on potential therapeutic targets and strategies for intervention [20].

This review aims to clarify the key mechanisms underlying AKI in CLD, highlighting the most relevant clinical presentations and management strategies. By emphasizing the importance of early intervention and targeted therapies, clinicians can improve outcomes for patients suffering from this complex interplay between pulmonary and renal dysfunction.

## METHODS

We searched for all published observational studies, randomized trials, systematic reviews, and metanalysis, from inception to 4 May 2024. PubMed database was searched using the following MeSH terms: (“chronic lung disease” OR “chronic respiratory failure” OR “chronic obstructive pulmonary disease” OR “asthma” OR “interstitial lung disease” OR “obstructive sleep apnea syndrome”) AND (“chronic kidney disease” OR “acute kidney injury” OR “kidney dysfunction” OR “kidney dysfunction” OR “kidney injury” OR “kidney damage” OR “kidney disease”). We eliminated cases that were published more than once by combining the words and cross-referencing the articles. We did not adhere to a strict methodological approach for the selection technique because the narrative nature of this review. We therefore screened the retrieved articles and their references for possible inclusion. Throughout this review, “kidney” will be used instead of “renal.” This aligns with the preferred terminology in most US-based medical journals.

## PATHOPHYSIOLOGICAL FACTORS CONTRIBUTING TO AKI IN CHRONIC LUNG DISEASE

Chronic acid–base disorders are common in patients with CLD, manifesting with stable hypoxemia and frequent hypercapnia. Hypercapnia results from inadequate ventilation, leading to an accumulation of carbon dioxide (PaCO_2_) in the bloodstream, which subsequently causes respiratory acidosis [21]. The primary causes of hypercapnia include obstructive lung disease, respiratory failure, and central nervous system depression, all of which impair gas exchange and CO_2_ clearance [21].

**Figure 1:**
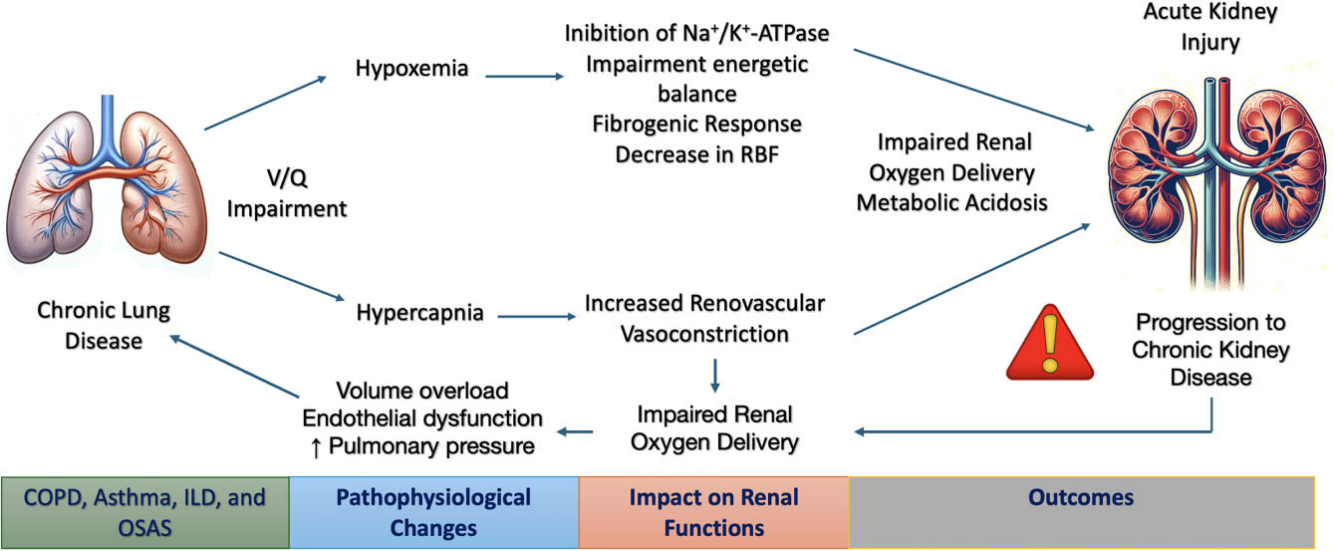
Pathophysiological pathways from CLD to AKI. This schematic illustrates the pathophysiological sequence linking CLD to AKI, with potential progression to CKD. The diagram highlights how hypoxemia and hypercapnia, resulting from ventilation/perfusion (V/Q) mismatch, reduce renal blood flow (RBF) and increase renovascular resistance, leading to impaired renal oxygen delivery and metabolic acidosis. Additional mechanisms, including pulmonary hypertension, endothelial dysfunction, and volume overload, further exacerbate renal congestion and oxygen imbalance, promoting sustained kidney damage and chronic injury.

In response to elevated PaCO₂, the kidneys increase bicarbonate reabsorption. In chronic conditions, the compensatory response is ∼+3.5 mEq/l of HCO₃⁻ for every 10 mmHg rise in PaCO₂. In acute settings, however, the compensation is limited to ∼+1 mEq/l per 10 mmHg increase due to reliance on non-bicarbonate buffering systems. However, during acute exacerbations of CLD, this compensatory mechanism is less effective, increasing only 1 mEq/l of bicarbonate per 10 mmHg rise in PaCO_2_ due to the reliance on hemoglobin and other non-bicarbonate intracellular buffers This limited compensation can exacerbate acid–base imbalances during respiratory failures, further contributing to kidney dysfunction [22]. In such hypoxemic conditions, while increased cardiac output might initially help mitigate effects, severe episodes often disrupt these compensatory mechanisms, leading to significant tissue hypoxia. This is especially critical in the renal medulla, particularly the outer medulla that is especially susceptible to hypoxic injury due to its relatively low oxygen tension combined with high metabolic demands for active sodium reabsorption [23]. These differences significantly influence the kidney's vulnerability during sustained hypoxemic conditions. Such vulnerabilities are exacerbated during acute crises where the demand for oxygen may rise without sufficient increases in cardiac output, potentially leading to AKI and further kidney dysfunction. Persistent hypoxemia and hypercapnia, from both acute exacerbations and chronic changes in blood gases, can severely impact kidney function by inducing physiological strains that the kidney system might struggle to accommodate, underscoring the importance of managing respiratory conditions to mitigate kidney damage [24]. The kidney cortex receives 20% of the cardiac output, while the kidney medulla receives only 10% of the total blood flow [25]. A low medullary blood flow is necessary to preserve medullary osmotic gradients for urinary concentration but makes the medulla more prone to impaired oxygen delivery and subsequent injury [26]. Insufficient kidney oxygenation determines inhibition of the sodium/potassium (Na^+^/K^+^)-ATPase, consequently leading to a downregulation of tubular transport and reduction of solute delivery for tubular reabsorption [17]. Various physiological studies show that hypoxemia (arterial oxygen saturation [SaO_2_] ∼83%–87%) leads to a reduction of kidney blood flow in a dose-dependent manner and an increase in filtration fraction with normal or slightly reduced estimated glomerular filtration rate (eGFR) [20, 27, 28]. Chronic hypoxemia typically observed in patients with CLD induces a loss of peritubular capillaries and activation of fibroblasts with a fibrogenic response [29]. The diffuse fibrosis determines a decrease in oxygen diffusion to tubular and interstitial cells, which causes mitochondrial dysfunction and persistent energy deficit in a vicious cycle leading to progressive kidney damage [19] (see Fig. 1, pathophysiological pathways from CLD to AKI).

By contrast, hypercapnia causes an increase in renovascular resistance with a decrease in kidney blood flow, presumably through activation of sympathetic tone, as reflected by the increase in the circulating levels of norepinephrine [20, 30, 31]. In addition, several studies have described a loss of kidney vasodilatory responses to various stimuli in the setting of hypercapnia (e.g. l-arginine [32] and protein loading [33]). Acute changes in PaCO_2_ levels might have a more dominant role than oxygen levels in determining renovascular resistance [20]. The decrease in kidney blood flow during hypoxemia and/or hypercapnia causes sodium retention with an imbalance of body water and the development of edema in the presence of normal cardiac output [34]. This event is also supported by alteration in the renin-angiotensin-aldosterone axis and the arginine–vasopressin system. Particularly, under hypoxemia and/or hypercapnia, decreased kidney blood flow triggers renin release from the kidneys, which activates angiotensin II to increase blood pressure and stimulates aldosterone secretion, thereby enhancing sodium and water reabsorption and leading to fluid retention. Moreover, pre-existing conditions such as obesity and tobacco smoking have been shown to intensify systemic inflammation and oxidative stress, further impairing renal endothelial integrity [5]. In patients with underlying CKD, this combination worsens renal perfusion and accelerates tubular injury. These overlapping risk factors reinforce the multifactorial nature of AKI in the context of CLD [5].

Concurrently, elevated plasma osmolality from these conditions prompts the hypothalamus to release arginine vasopressin, increasing water reabsorption and further augmenting fluid retention. The combined action of angiotensin II and vasopressin in CLD exacerbates fluid retention, heightening the risk of edema formation [34]. The concomitant rise in filtration fraction increases the oncotic pressure in peritubular capillaries, which enhances the reabsorption of sodium and water in the proximal tubule. This mechanism promotes intravascular volume expansion and contributes to sodium and fluid retention, particularly in the setting of preserved or elevated glomerular filtration pressures.

Positive intrathoracic pressure, generated during mechanical ventilation, can significantly influence renal hemodynamics. High positive airway pressures, especially during invasive mechanical ventilation, reduce venous return depending on respiratory system compliance, increase right ventricular afterload, lower cardiac output, and raise central venous pressure, thereby impairing renal perfusion [35]. Additionally, elevated intrathoracic pressures can directly increase renal venous pressure, reduce the transrenal pressure gradient, and promote interstitial edema, contributing to the so-called “kidney compartment syndrome” [35].

By contrast, non-invasive ventilation (NIV), when used appropriately, has a more favorable hemodynamic profile. NIV improves oxygenation and reduces respiratory muscle work without imposing the same level of intrathoracic pressure as invasive mechanical ventilation. This can lead to more stable cardiac output, less central venous congestion, and potentially better renal perfusion [36]. While direct evidence on renal blood flow or glomerular filtration during NIV is limited, observational data and physiological reasoning suggest that NIV may reduce the incidence of AKI, particularly in patients with underlying CKD or borderline hemodynamics. These effects are likely mediated by improved gas exchange, reduced sympathetic activation, and avoidance of intubation-related hemodynamic instability [9, 37, 38]. Further studies are needed to quantify the direct impact of NIV on renal perfusion, but its protective potential, especially in the setting of CLD and incipient renal impairment, should be considered in clinical decision-making.

In addition, acute hypoxemia and hypercapnia markedly increase pulmonary vascular resistance and may lead to right ventricular dysfunction and increased central venous pressure [39], which may further decrease kidney perfusion pressure and oxygen delivery by inducing kidney venous congestion and increase kidney interstitial pressure (kidney compartment syndrome) [40]. An often-overlooked but crucial component in the lung–kidney interplay is the cardiovascular system. CLDs, particularly when complicated by pulmonary hypertension, can lead to right ventricular dysfunction and elevated central venous pressure. This results in renal venous congestion, impaired glomerular filtration, and progressive renal injury, mechanisms that are characteristic of type 3 cardiorenal syndrome, in which primary pulmonary dysfunction leads to secondary heart and kidney involvement [41]. The bidirectional nature of this syndrome underscores the need for an integrated approach to organ interactions in critical illness [41].

**Figure 2:**
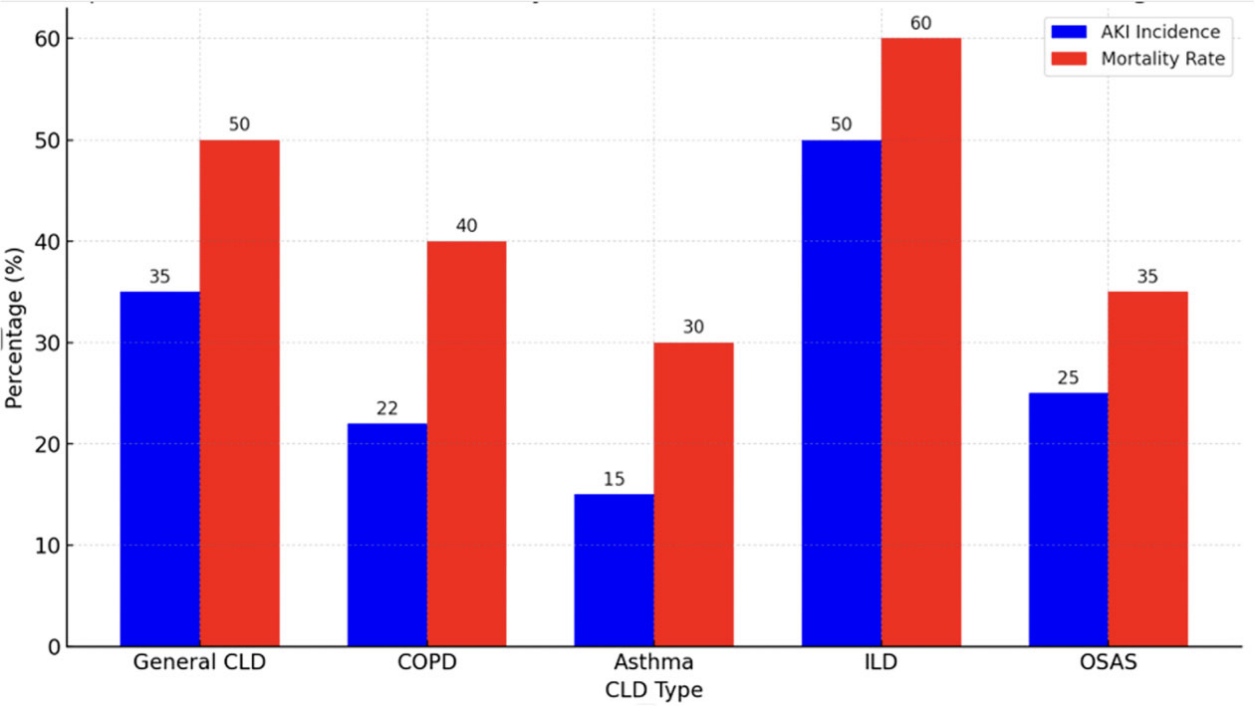
Comparative incidence and mortality rates of AKI in patients with CLDs. This figure presents data from published studies reporting the incidence of AKI and in-hospital mortality in patients hospitalized for acute exacerbations or respiratory decompensation of CLDs, including general CLD [113], COPD [16], asthma [114], ILD [89], and OSAS [115]. The blue bars indicate the proportion of patients who develop AKI, whereas the red bars represent the mortality rates for these patients, highlighting significant risks and outcomes associated with kidney complications in these conditions. Note: All values refer to AKI incidence and mortality during hospitalization for acute events. Data are derived from independent studies with varying definitions, settings, and populations. The figure is intended to provide an illustrative overview, not to imply direct comparability. Differences in characteristics—such as age, comorbidities, and baseline severity—may influence outcomes and limit direct comparability.

Kidney auto-regulation may become impaired due to decreased availability of nitric oxide, which impairs tubulo-glomerular feedback [42]. Figure 2 illustrates the AKI incidence and mortality rates associated with general CLD, chronic obstructive pulmonary disease (COPD), asthma, interstitial lung disease (ILD), and obstructive sleep apnea syndrome (OSAS). The data shown were extracted from independent studies with differing patient populations, and are therefore not directly comparable but serve an illustrative purpose.

## CLINICAL PRESENTATION

The common clinical pictures of CLD admitted to the ICU and the kidney function in critically CLD patients is described in the following section (refer to Table 1, pathophysiological factors and clinical presentations of CLD in ICU setting).

**Table 1:** Pathophysiological factors and clinical presentations of CLD in ICU settings.

CLD type	Pathophysiological factor	Effect on kidney function	Clinical presentation	Kidney implications	References
COPD	Hypoxemia and hypercapnia	Reduced KBF and GFR; increases renovascular resistance	Hypoxemia, hypercapnia, acidosis	Increased risk of AKI, especially during exacerbations; chronic hypoxemia leading to CKD	[24]; [20]; [30]; [31]
Asthma	Hypoxemia	Reduced RBF and glomerular filtration rate	Reversible airflow obstruction, respiratory acidosis	AKI risk during severe exacerbations due to systemic steroid use and hypoxemia	[74]; [80]
ILD	Chronic hypoxemia	Impairs oxygen delivery, leading to energy deficits and tubular injury	Chronic inflammation, fibrosis, restrictive ventilatory defect	High incidence of AKI during exacerbations; CKD in advanced stages	[81]; [83]
OSAS	Intermittent hypoxemia	Chronic hypoxemia leading to glomerulosclerosis and CKD	Intermittent hypoxemia, increased sympathetic activity	Chronic hypoxemia can lead to pulmonary hypertension, right ventricular failure, and systemic hypertension exacerbating kidney function decline	[85]; [116]

Abbreviation: KBF: kidney blood flow rate.

### Chronic obstructive pulmonary disease

COPD currently ranks as leading cause of death worldwide and is projected to rise to third place by 2030 without targeted interventions [43, 44]. Its stands at an estimated 13.6% among urban populations and 9.7% among rural communities [45]. COPD often coexists with conditions such as ischemic cardiovascular disease, heart failure, diabetes mellitus, and frequently undiagnosed CKD [46, 47]. During acute exacerbation of COPD, airflow limitation alters the Ventilation-perfusion (V/Q) ratio, leading to hypoxemia and hypercapnia, which can precipitate kidney injury, especially in the presence of pre-existing CKD and other comorbidities [31]. In patients with COPD undergoing mechanical ventilation, hypercapnia can lead to kidney vasoconstriction and decreased kidney blood flow, potentially exacerbating kidney hypoperfusion and increasing the risk of AKI [13]. Studies indicate that COPD exacerbations necessitating mechanical ventilation significantly elevate the risk of AKI, due to hemodynamic instability and systemic inflammation [48]. This underscores the importance of monitoring and managing both respiratory and kidney functions meticulously during COPD exacerbations to mitigate the potential for severe kidney complications. Indeed, patients with COPD demonstrate a notably higher incidence of AKI and prevalence of CKD compared to age-matched and gender-matched counterparts [49]. Additionally, COPD patients frequently experience peripheral skeletal muscle wasting and weakness, potentially leading to an underestimation of CKD due to relative decreases in serum creatinine concentrations [50, 51].

Factors contributing to muscle wasting in COPD include hypoxia, chronic acidosis, elevated levels of pro-inflammatory mediators, and treatment with steroids [52]. During exacerbations, AKI incidence is relatively high, and its presence serves as a prognostic indicator of poor outcomes [16]. Trudzinski *et al.* [53] examined the link between acid–base balance, blood gases, kidney function, and respiratory function in stable COPD patients, using the COSYCONET cohort data. The authors found that changes in acid–base balance, lung, and kidney function correlate with exacerbation risk in COPD. Base excess indirectly predicts exacerbation risk through its association with eGFR, lung function, and PaCO_2_. Reduced eGFR has been independently associated with a higher risk of acute exacerbations in patients with CLD, even after adjusting for the effects of pharmacological treatments. These findings highlight the importance of considering acid–base balance and kidney function in assessing exacerbation risk in stable COPD patients. In a recent study serum levels of cystatin C and beta-2 microglobulin (β2-MG) were shown to increase progressively with hypoxia severity in 106 elderly patients with acute exacerbations of COPD, compared to 60 healthy controls. These biomarkers, associated with kidney dysfunction, showed significant correlations with inflammatory markers and kidney function indicators, highlighting their potential for early detection of kidney impairment in this patient group.

Muscle wasting in COPD results from factors such as hypoxia and chronic acidosis, which limit energy production, and depress muscle protein synthesis [50]. Elevated pro-inflammatory mediators, steroid treatment, pre-existing impaired kidney function, polytherapy, advanced age, and low body mass index are associated with muscle wasting and increase the risk of AKI in COPD patients [16, 52]. Additionally, COPD contributes to airway and systemic inflammation, leading to a high prevalence of endothelial dysfunction and albuminuria. Conversely, the nocturnal use of bilevel-positive airway pressure ventilation is linked to reduced endothelial dysfunction, albuminuria, and preservation of kidney function [54]. During acute exacerbations of COPD, AKI is prevalent and associated with poor outcomes [16]. Moderate oxygen supplementation alleviates hypoxia but requires careful monitoring to prevent hypercapnia [55, 56]. NIV is preferred over invasive mechanical ventilation because, by maintaining a lower intrathoracic pressure, it reduces the risk of complications associated with prolonged use of high positive pressure and maintains kidney function [2, 5, 43]. Prompt initiation of pharmacotherapy with bronchodilators and β2-agonists optimizes oxygenation and protects kidney tubules [58, 59]. Corticosteroids, though beneficial in animal models, must be cautiously dosed during kidney replacement therapy due to the risk of hypophosphatemia exacerbation [60–62]. Although corticosteroids may contribute to fluid retention and electrolyte imbalances, some evidence suggests they may also exert renal protective effects in selected clinical contexts. For instance, early corticosteroid therapy in critically ill patients with severe coronavirus disease 2019 (COVID-19) has been associated with a lower risk of AKI in the intensive care unit setting [63, 64]. Furthermore, corticosteroids may mitigate the risk of antibiotic-induced acute interstitial nephritis (AIN), a relevant cause of AKI in patients receiving empirical antimicrobial treatment [65].

Roflumilast, a phosphodiesterase (PDE)-4 inhibitor, is recommended for severe COPD cases without kidney dose adjustments [66, 67]. Antibiotic treatment for COPD exacerbations should account for bacterial resistance patterns and individual patient factors while considering the potential for nephrotoxicity. Similarly, antibiotics and antiviral medications may pose a risk of direct kidney damage, necessitating dosage adjustments in cases of kidney impairment. The mechanisms of nephrotoxicity include direct tubular cell damage [68], leading to conditions such as acute tubular necrosis, osmotic nephrosis, and crystalline nephropathy, as well as damage to interstitial cells, potentially causing acute interstitial nephritis [68, 69]. If kidney function declines, antibiotic dosages should be decreased, and adjustments in frequency may be necessary during kidney replacement therapy. Care must be taken to avoid both excessive dosing, which can contribute to antimicrobial resistance [70], treatment failure, and underdosing. Antiviral drugs, such as cidofovir, adefovir dipivoxil, tenofovir, and acyclovir, may exert kidney tubular toxicity, leading to prolonged elimination half-lives in patients with kidney impairment [71].

## ASTHMA

Asthma is a widespread chronic respiratory condition marked by airway inflammation, bronchoconstriction, and increased airway responsiveness [72]. It is primarily known for causing reversible airflow obstruction [72]. The chronic nature of asthma and its recurrent exacerbations can have a notable impact on kidney function, although through mechanisms different from those observed in COPD or ILD. Severe asthma exacerbations requiring mechanical ventilation can exacerbate kidney stress due to systemic inflammation, hypoxemia, and the use of medications such as systemic corticosteroids, which are known to affect fluid and electrolyte balance [73]. This can lead to a cascade of kidney challenges, including vasoconstriction and reduced kidney perfusion, potentially culminating in AKI. During severe exacerbations, asthma patients commonly endure significant respiratory distress, hypoxemia, and respiratory acidosis [74]. These conditions lead to heightened sympathetic nervous system activity that causes kidney vasoconstriction. Consequently, there is a reduction in kidney blood flow and GFR [75], factors that predispose individuals to AKI during these critical episodes. Furthermore, the systemic use of corticosteroids, integral to managing exacerbations, can disrupt fluid and electrolyte balances, potentially worsening kidney dysfunction [58].

The persistent inflammation associated with asthma may lead to endothelial dysfunction and systemic inflammatory responses, adversely affecting kidney health [76]. Additionally, long-term asthma is often linked with comorbidities such as hypertension and cardiovascular diseases, which can further impair kidney function [76, 77]. Managing exacerbations in asthma requires meticulous attention to kidney function, especially in patients with existing kidney impairments or those at risk for AKI [78]. Optimizing asthma treatment involves the careful use of corticosteroids and bronchodilators. Monitoring kidney function parameters is crucial to prevent kidney complications [79]. This approach includes adjusting medication dosages and regimes to avoid exacerbating pre-existing conditions.

Understanding the complex relationship between asthma and kidney dysfunction is vital for comprehensive patient management [78]. This understanding underscores the importance of a multidisciplinary approach that addresses both respiratory symptoms and kidney health, ensuring holistic patient care (see Table 2).

**Table 2:** Impact of CLD management strategies on kidney function.

Management strategy	Pulmonary benefit	Impact on kidney function
NIV	Reduces work of breathing and improves oxygenation	May decrease kidney hypoxia but can also increase intrathoracic pressure, reducing renal perfusion
Bronchodilators and corticosteroids	Reduce airway inflammation and improve airflow	Corticosteroids can cause fluid retention and electrolyte disturbances, increasing AKI risk
Optimized fluid management	Prevents pulmonary edema and improves oxygenation	Balances volume status, preventing fluid overload and worsening kidney congestion
Diuretics	Reduces pulmonary congestion and improves breathing	Can cause electrolyte imbalances and pre-renal azotemia if overused
Pulmonary rehabilitation	Improves respiratory muscle function and endurance	Indirect benefit by reducing hospitalizations and risk of AKI from acute exacerbations
Oxygen therapy	Corrects hypoxemia and reduces work of breathing	Excessive oxygenation can lead to hypercapnia, worsening acid–base imbalances in CKD patients
Antibiotic and antiviral therapy	Manages respiratory infections to prevent exacerbations	Must be adjusted for renal function to avoid nephrotoxicity
Kidney replacement therapy	Supports organ function in critically ill patients	Essential in severe AKI cases to manage volume overload and toxin clearance

While asthma exacerbations typically do not elevate AKI risk as significantly as COPD, the potential for kidney impact exists and necessitates careful clinical oversight [80]. Vigilant management of asthma, particularly during exacerbations, and a strategic focus on maintaining kidney health are paramount in minimizing complications and improving overall patient outcomes.

## INTERSTITIAL LUNG DISEASE

ILD encompasses various pulmonary disorders characterized by chronic inflammation, fibrosis, and interstitial remodeling, often leading to a restrictive ventilator pattern on spirometry. Idiopathic pulmonary fibrosis is the primary cause of ILD accompanied by sarcoidosis, autoimmune/connective tissue diseases (e.g. rheumatoid arthritis, systemic lupus erythematosus, systemic sclerosis), pneumoconiosis, vasculitis, drug exposure (including amiodarone, chemotherapeutic agents, intravenous illicit drugs), and radiation [81]. Comorbidities associated with ILD include cardiovascular disease (20%), hypertension (15%), diabetes mellitus (11%), depression (11%), gastroesophageal reflux disease, and venous thromboembolism, often leading to pulmonary hypertension and right ventricular failure at advanced disease stages [82]. AKI significantly affects ILD mortality rates, escalating from 41% to 53.7% over 1 year [83]. During acute exacerbations of ILD, AKI occurrence reaches 50%, is independently associated with increased mortality (odds ratio of 10.6) [83].

In managing ILD, patients may require ICU support if gas exchange deteriorates rapidly [84]. Early trials of NIV should be initiated, although mechanical ventilation poses considerable risks, with mortality rates ranging from 47% to 89%, depending on ventilation method [36]. Invasive mechanical ventilation carries a higher infection risk. Limited research on AKI in mechanically ventilated ILD patients underscores the need for studies focused on managing and preventing kidney damage during ILD exacerbations [36].

The vasculitis pattern in ILD predisposes patients to a high risk of CKD, resulting from damage to lung and glomerular blood vessels [85, 86]. Acute exacerbations of ILD manifest as worsening dyspnea, new radiographic opacities, and hypoxemia, without evidence of other pulmonary conditions [87, 88]. In a recent study, restrictive lung dysfunction was found to correlate with lower GFR levels, particularly evident in advanced CKD stages. Individuals presenting cardiovascular disease, protein-energy wasting, and inflammation exhibited a heightened prevalence of restrictive lung disorder. These findings underscore the significant interplay between pulmonary and kidney function, emphasizing restrictive lung disorder as a prevalent complication among advanced CKD patients [89].

Although no curative therapies exist for most ILDs [90], antifibrotic agents such as Pirfenidone and Nintedanib have demonstrated efficacy in slowing disease progression and improving clinical outcomes in selected forms of progressive fibrosing ILDs beyond diopathic pulmonary fibrosis [91]. Treatment primarily relies on immunosuppressive drugs, albeit with nephrotoxicity and AKI risks [92, 93]. Corticosteroids, often adjuncts to immunosuppressants, require cautious administration due to potential kidney crisis [94]. Combining corticosteroids and immunosuppressants with *N*-acetylcysteine, known for antioxidant and vasodilatory properties, may offer symptomatic relief and slow disease progression [77]. Considering lung transplantation for all ILD patients is essential [94]. In summary, kidney function can be adversely affected in patients with ILD, leading to AKI, CKD, or exacerbation of existing kidney dysfunction. Screening patients before initiating therapy is crucial to rule out active infections or significant organ dysfunction that could potentially interfere with drug metabolism and excretion. This proactive measure ensures that the chosen therapy can be safely and effectively administered, minimizing risks and optimizing treatment outcomes [96]. Close monitoring, avoidance of nephrotoxic medications, and a multidisciplinary approach are key components of effective management in these patients.

## OBSTRUCTIVE SLEEP APNEA SYNDROME

OSAS is the predominant form of sleep-disordered breathing, affecting 2%–4% of the global population [97]. It is characterized by recurrent episodes of partial or complete obstruction of the upper airway during sleep. These episodes, which can occur numerous times per hour of sleep, lead to significant reductions in blood oxygen saturation, sleep fragmentation, and increased sympathetic nervous system activity, resulting in frequent awakenings, oxygen desaturation, and intermittent hypoxia [97]. OSAS is diagnosed using the apnea-hypopnea index (AHI), which quantifies these events per hour of sleep. A diagnosis is confirmed with an AHI of >15 events per hour regardless of symptoms, or >5 events per hour when symptoms are present. The relationship between OSAS and CKD is complex and likely multifactorial. While OSAS has been associated with an increased risk of kidney dysfunction, it is more probable that shared comorbidities, such as obesity, hypertension, and diabetes, play a central role in the development of CKD rather than OSAS itself being a direct causal factor. OSAS may contribute to kidney dysfunction through intermittent hypoxia, sympathetic activation, and systemic inflammation, which can exacerbate existing kidney disease rather than directly causing it. Unless compelling evidence can support a causal relationship, it is more accurate to frame OSAS as a contributing factor rather than a primary cause of CKD.

In the ICU, mechanical ventilation, particularly non-invasive methods such as CPAP, is crucial for managing severe OSAS episodes. These interventions help stabilize oxygen levels and reduce the strain on the cardiovascular system, which indirectly benefits kidney function by maintaining more stable hemodynamics [38]. However, the positive pressure from these devices can also affect cardiac output, potentially leading to reduced kidney perfusion and increased risk of AKI [98]. Multiple processes, such as changes in chemoreflex responsiveness, pharyngeal constriction from fluid overload, and buildup of uremic toxins, can cause CKD and OSAS. It is now becoming more well acknowledged that OSAS might hasten the deterioration of kidney function [99]. Potential mechanisms linking CKD and OSAS include Na^+^ and water retention with fluid overload, pharyngeal narrowing, and tongue enlargement with possible airway obstruction. Following CKD, metabolic acidosis is associated with hyperventilation and hypocapnia, which can increase chemoreflex sensitivity. Chemoreflex control and sleep respiratory control can be altered, thus PaCO_2_ reduces below apneic threshold causing apnea [100]. It has been reported a prevalence of CKD of 30.5% with mean eGFR in severe OSAS of 38.8 ml/min [101, 102]. OSAS-associated CKD can be further complicated by the presence of hypertension, diabetes, and old age. For this reason, researchers are exploring the possibility of increased CKD risk in non-diabetic, non-hypertensive OSAS patients [103]. Results indicate that AHI and the desaturation index were the only significant predictors at multivariate analysis for urine albumin-to-creatinine ratio and eGFR [104].

The need for ICU admission becomes critical when these chronic conditions acutely exacerbate. OSAS patients experiencing severe hypoxemic episodes or respiratory failure require immediate and intensive management strategies that often necessitate ICU care. The ICU setting provides advanced respiratory support, including invasive and non-invasive mechanical ventilation, which is crucial during severe exacerbations to stabilize patient conditions. Continuous positive airway pressure therapy, the first-line treatment for OSAS, is often initiated in the ICU to manage acute respiratory distress effectively and to prevent further complications, including severe hypoxemia and cardiac stress [86]. In addition to continuous positive airway pressure, alternative strategies such as mandibular advancement devices, positional therapy, and weight loss programs are considered to alleviate the symptoms of OSAS and improve long-term outcomes. Surgical interventions to remove obstructive tissue may also be necessary for some patients [86].

In the ICU, close monitoring and aggressive management of kidney function are imperative, especially given the high risk of AKI in these patients. Optimizing fluid and electrolyte balance, managing blood pressure, and adjusting medications in response to dynamic kidney function are key components of ICU care. Enhanced kidney support, potentially including kidney replacement therapy, may be required depending on the severity of kidney injury [105].

## LUNG TRANSPLANTATION

CKD is a frequent and serious complication following lung transplantation, affecting up to 50% of recipients within five years [106]. The pathogenesis of post-transplant CKD is multifactorial and includes nephrotoxicity from calcineurin inhibitors, such as tacrolimus and cyclosporine, commonly used for immunosuppression [107]. These agents cause renal vasoconstriction, tubular injury, and chronic interstitial fibrosis. Additional contributors include perioperative hemodynamic instability, AKI during ICU stay, and underlying risk factors such as diabetes or hypertension [108]. Furthermore, pulmonary hypertension and right ventricular dysfunction, common in end-stage lung disease may lead to chronic renal venous congestion, compounding kidney damage [109]. Exposure to nephrotoxic antibiotics and volume depletion also increases the risk. Post-lung transplant CKD is associated with worse long-term outcomes, and in selected cases may necessitate kidney transplantation [110]. Monitoring renal function and tailoring immunosuppression are essential to prevent further decline in kidney health.

## CONCLUSIONS

This review underscores the complex interdependence between CLD and kidney function, particularly highlighting the mechanistic pathways that lead to AKI during exacerbations of pulmonary conditions. It explains how common elements of CLDs, such as hypoxemia and hypercapnia, directly contribute to kidney dysfunction by impairing kidney blood flow and increasing renovascular resistance These altered blood gas parameters foster an environment prone to kidney damage, which can exacerbate the severity of lung diseases, thus propagating a cycle of declining organ function. Management of such exacerbations, particularly in critical care settings, necessitates interventions such as continuous positive airway pressure to mitigate the effects on the kidneys. Finally, the presence of modifiable shared risk factors such as smoking and obesity, in addition to CKD, should not be overlooked. Their contribution to both pulmonary and renal dysfunction underscores the necessity of multidisciplinary interventions that go beyond acute care, incorporating lifestyle modification, smoking cessation programs, and metabolic control to prevent long-term organ damage. Future research is essential to further delineate the relationship between pulmonary pathologies and kidney outcomes, with a focus on developing targeted treatments that address these interconnected issues. Effective management strategies must encompass a multidisciplinary approach, focusing on fluid management, blood pressure control, and the avoidance of nephrotoxic agents to prevent the progression of AKI to CKD. In rare but clinically significant cases, combined lung–kidney transplantation may be considered in patients with end-stage CLD and coexisting advanced CKD. This strategy may be appropriate when both organs exhibit irreversible dysfunction, and the patient is eligible for dual transplantation [111]. Typical candidates include individuals with idiopathic pulmonary fibrosis, advanced COPD, or cystic fibrosis accompanied by a GFR persistently <30 ml/min/1.73 m², dialysis dependence, or rapidly progressive kidney disease [112]. Although data are limited, recent reports suggest that outcomes for selected patients undergoing combined transplantation can be comparable to those of isolated lung recipients, provided that a rigorous multidisciplinary assessment is performed before listing [112].

Through such focused efforts, it is possible to improve the prognosis and overall health outcomes for patients suffering from these interrelated conditions.

## Data Availability

No new data were generated or analyzed in this study. Data sharing is not applicable to this article as no datasets were created or used.
